# On the Efficacy of Belladonna as a Remedy in Pertussis

**Published:** 1852-11

**Authors:** Hiram Corson

**Affiliations:** Corson Co., Pa.


### SELECTIONS.

From the American Journal of the Medical Sciences.

*On the efficacy of Belladonna, as a Remedy in Pertussis*, by HIRAM CORSON, M. D., of Corson Co., Pa.

I HAD been engaged in practice six years, when the essay of Dr. Jackson, of Northumberland, "On the Efficacy of Belladonna in Whooping-cough," was published in the *American Journal of the Medical Sciences*, (August No., 1834.) My experience in pertussis had satisfied me that all the remedies in common use—those recommended by writers upon the diseases of children—were almost useless. Children affected in the Winter, continued to cough, and strangle, and suffer, for many weeks, with scarcely a perceptible amendment; and even when convalescence did commence, a long time elapsed before complete recovery. It was painful to visit, and mortifying to prescribe for, those afflicted with this malady; and so apparent was our inefficiency, that many families did not seek the aid of a physician at all; and, if urged by their friends or neighbors to do so, justified themselves by saying that "every body knows the doctors can do nothing for whooping-cough, it will have its time." I had arrived at nearly the same conclusion previous to the publication of Dr. Jackson referred to.

In the winter of 1844-5, whooping-cough prevailed in the region above Norristown. It had not visited that neighborhood for many years previously, so that nearly all the children at the schools were liable to the disease; and, with few exceptions, they became affected, so that the schools were nearly broken up. My brother had four little boys severely affected, and, as I was desirous to try Dr. Jackson's remedy, I read his essay to my brother, and urged the use of the belladonna. They were strong, healthy little boys, and had the disease severely. I do not now recollect the dose that we used, but they were put fairly under the influence of the medicine, every day, by repeating the dose every three hours. In one week they were all well, and returned to school, while the children of many of the neighbors suffered with the cough for several weeks afterward.

These cases occurred a few miles beyond the limits of my practice, and I saw no more of the disease until it occurred in our own region in the following year.

January 14, 1836, Mr. S. called upon me to visit his son, two years old. He said the child had been laboring under whooping-cough for several weeks, and he did not think it could live more than a few days, for it was coughing up *matter*, but he thought he had better come for me, lest the people should make a fuss about his not getting a doctor. I told him of the new remedy; he agreed to its use; we gave it for one week, when the child was perfectly cured of the cough, though he remained weak for a short time.

A brother-in-law of Mr. S., who resided many miles distant, came to me for some of the medicine for his child, who, he said, "had the whooping-cough so badly that they thought it would strangle to death every night."

From the little experience that I had had with the remedy, it seemed to me that the dose recommended by Dr. Jackson, viz., one grain to a child two years old, was too large. I therefore concluded to begin with children under one year, with the sixteenth or eighth of a grain, every two hours, and to increase a little every day, until the proper dose was reached. With this view, I put eight grains of extract of belladonna into one ounce of water. Nine drops of this solution contained just an eighth of a grain of the extract. Of this I directed him to give his child nine drops every two hours, until the pupils were dilated, face flushed, mouth dry, and vision confused. If these effects were not produced the first day, then to increase the dose three drops daily. I was informed that the child was entirely cured in a week.

From this time it was used in all cases that presented, and in every instance in which it was properly administered, the cure was speedy.

In 1840, the disease prevailed over my district, and I used the belladonna in hundreds of cases, with great relief in nearly all.

There were some cases, however, in which I saw no good produced; in these, the medicine did not seem to produce any effect. It is probable that the fears of the parents did not allow them to use the remedy properly. The parents were always informed of the nature of the medicine, its mode of action, and the change effected upon the pupil of the eye. Some became much alarmed, and strange stories were circulated, that I used a "medicine that was a poison, and swelled the eyes." But the relief obtained by those who used it was so great, the cures so rapid, and the medicine so pleasant to take, that one after another came to try it, until it became the only thing that was thought worth using.

I was not alone, at this time, in its use. Dr. George Thomas, and Dr. Wm. Corson, of Norristown, also prescribed it.

In order that the fears of our patients should be allayed, we always desired that the first few doses should be so small as to produce no marked effect, and thus, the child not getting so quickly under its influence, the cure was somewhat retarded, but generally occurred within two weeks; when, judging from cases which afterwards occurred, I doubt not they might have been produced in less than one week.

I will mention a few cases which were quite remarkable, and certainly should aid us in forming a judgment of the nature of this disease. I take the following cases from my note-book :—

A girl, six years old, has had the cough three weeks; paroxysms recur, the parents think, about forty times in twenty-four hours, and are very violent. Ordered one-fourth of a grain of the extract of belladonna every two hours, until the characteristic symptoms are produced. In about fifteen minutes, the face was much flushed, there was ringing in the ears, and croupy sound in breathing.—After those symptoms passed off, a like dose was given; the same symptoms, but milder in degree; medicine repeated whenever the influence had nearly passed off. On the fifth day there was not a trace of the disease left. This child had always been liable to croup, and had suffered some violent attacks.

Another child of the same family, a little boy, two years old, had been left until the disease had continued two weeks, and had acquired the real whoop. He had to be caught up many times through the night, for fear he would suffocate. Began by directing for him one sixth of a grain every two hours. In a few minutes after the first dose was taken, his face became much flushed, pupils dilated, and symptoms of croup followed of such severity that I was sent for in much haste. It was 9 A. M. The case seemed just like one of spasmodic croup. I administered hive syrup, vomited him freely, with much relief, and before the next morning the croupy sound had disappeared; there were but five coughing spells through the night. Next morning another dose was given; the croupy symptoms were produced, but not so severely. The flush of face, enlargement of pupils, and croupy sound, passed off before bed-time, and there was no cough worthy of notice through the night. A third dose was given, and from this time, no one could have told that the child had ever had whooping-cough.

Those cases occurred in June, 1840, and never before had I seen such prompt effects from the medicine.

In August of the same year, called to C. W.'s son, aged nine months. Has had the disease two weeks. His mother thinks he coughs every fifteen minutes, night and day. Ordered nine drops of the solution every three hours; increase three drops daily. In one week the cough had diminished to about four or six times in twenty-four; from being sick, restless, and moaning, it is now well and lively.

J. W.'s child, aged one year, coughs badly, the mother says, " from noon until next morning; takes no suck, and notices nothing scarcely." This child took the medicine, but had so much difficulty in respiration, from the secretion into the bronchial tubes, that we gave ipecacuanha every day to clear the passages. In three weeks, it was perfectly well.

*August* 21. A boy, aged two and a half years. The mother told me an older child had taken the disease in April, that it was still coughing much, and its health was much broken; that she had been so worried with it during so long a time, being much disturbed at night, that she thought if this one was to go through a similar course, she could not stand it. And added, "that *this* child, who has now had it six weeks, is *worse* than the other ever was." At 10 A. M., I directed nine drops, (⅛ gr.,); in a few minutes, the face flushed, the child seemed much excited, and, after a short time, fell into a profound sleep, which somewhat alarmed the mother. He continued to sleep with little interruption, until next morning; but during all this time, to our utter astonishment, he never once coughed. Next day we gave him seven drops; effects not so severe. Third day gave six drops; from this time he was perfectly well.

*Sept.* 1. G. R.'s child, aged *two months.* The mother says: "Has had the disease badly three weeks, coughing and strangling by night and day; it can't live without some help soon, and I am determined to try the medicine." Began with one drop every 3 hours; increased one drop every day, until it took five at a dose. By this time it was almost well. Had no more trouble with it.

*Sept.* I. J. A.'s child, aged four months, very bad with cough. Gave four drops every three hours; increase one drop every day, if necessary. In four days, comparatively well.

*Oct.* 4. J. T.'s child, aged three months, female, coughs and whoops badly. Ordered five drops every three hours; first dose given while I was present. I immediately left for home. In about fifteen minutes after I reached it, a messenger arrived in great haste, and said the child had fits. I returned, and found it partially recovered from what they described as "a fit, or strange spell; it seemed so wild, and looked so queer, and appeared as though it could not get its breath good." It slept well that afternoon and night. Gave the medicine for a few days, in less doses; and in less than a week there was not a vestige of the disease left.

*Oct.* 15, 1840, the following note was made: I am now giving the belladonna to children all through this region, and in no case has it failed to cure, in from three days to three weeks. The cures seem perfect. The disease has occurred this season in families, the older children of which had been cured by the belladonna, four years ago. In no instance has one of those taken the disease a second time.

Persons living out of my range of practice, have often applied to me to come and see their children. My rule was to offer them a portion of the extract, with a letter to their physician, directing him how to use it. This they sometimes declined; and then I gave them the solution, with directions for its use. A young man came to me, and stated that his sister's children had the whooping-cough so badly that one of them, three years old, was entirely confined to bed; and the other, of five years, was hardly able to be about. He wished me to give him medicine for the former; for the latter, they were trying a remedy from Philadelphia. In 3 days, he returned and wanted medicine for the latter, saying that the former was nearly well. Four days after, he came again, to get some for a neighbor; and said he considered his sister's children well. I never saw them. Every person was told what the medicine was, and how to use it.

In the Summer of 1847, I was called to see a little boy, four years old, and his sister of two years. I cannot truly describe the condition of those little sufferers. Their faces, bodies, and limbs were bloated; they were sallow and bloodless-looking; frequent pulse after fits of coughing, with hurried respiration, mucous rhonchus, and excessive general distress and debility. The cough was frequent, prolonged, and exhausting; and in one of them, was accompanied by discharges from the rectum, during every paroxysm. A younger child, of nearly a year old, lay dead in the house, from the same disease; although a most eminent physician, aided by the best means that wealth could command, had been in constant attendance. Nothing had done any good for any of these three children. The belladonna and carb. ferri were given to the two to whom I was called, and in ten days they were well. The result was truly astonishing.

A man came from Chester County to get some medicine for whooping-cough. I gave him some to take to his physician, wrote my directions for using it, and referred him to Dr. Jackson's communication, in the *Medical Journal.* In a few months I met the same man, and asked him how his child was; he said that he did not know that the medicine did any good; the child was a long time getting well; that the doctor had called at his house recently, and told them that he had killed three children with it, and had ceased to prescribe it. I asked: Is he intemperate? He replied in the affirmative. A few months afterward, he died of delirium tremens.

In November, 1850, the disease again appeared in this region, and I used the belladonna freely, with good effect. I will briefly mention a few cases.

Three children, of three, five, and seven years of age, coughing badly; began the youngest with nine drops, the others more; increased rapidly until perceptibly affected. Well in ten days.

*Nov.* 19. M. R.'s son, three years old, has taken the medicine for one week. Mother thinks him nearly well. 25th. Is quite well. A younger child afterwards took the medicine.

Three children, eldest five years old, youngest four months, took the medicine so as to be under its influence daily; well in one week. Scores of such cases might be offered, but it is unnecessary.

I will mention one of a different character.

S. S.'s child, boy, five years old, coughs badly, strangles much.

Nov. 13, began with ten drops every three hours. 17th. Takes thirty-five drops every three hours; no relief. Ordered it every two hours, and increased freely. 21st. Takes ninety drops every two hours; produces none of the characteristic effects of belladonna. I continued this medicine, in teaspoonful doses, for several days longer; but with little apparent effect upon the cough.

I cannot believe that the parents deceived me in their account of the case. The other children were also taking it, and with relief; while this one suffered so much as to be the greatest cause of anxiety to them. Nor was there any other disease. It was a pure whooping-cough. It is true that I prescribed it in very many cases, in which it did but little good; but that arose from the inefficiency of the administration.

*Jan.* 15, 1851. I prescribed the belladonna for four children in one family. The youngest was nearly two years old, and suffering somewhat from the irritation of teething. It took one dose of nine drops the first day; another dose next day; and about ten o'clock on the third day, got a severe fit. I was sent for in much haste. When I came, the mother said, "Doctor, you have killed my child with that stuff. I'll never give another drop of it to any of my children." As I knew the family well, I reminded her that others of them had had fits while teething. She said that not one of them, seven in number, had escaped having fits while teething. The child soon recovered from it, and yet the mother continues to say that she does not know that it was the medicine that produced the fit, but she would never give it. There was not the least evidence of the child being affected by the belladonna.

I mention this case to show how it is that there is a fear kept alive among the people, in relation to this medicine. Such cases are noised abroad. If any thing at all should happen to a child which is taking belladonna, it is charged to the medicine; hence physicians are reluctant to assume the responsibility of giving it, when they can pursue the old plan, which is without risk.

During the last seventeen years, I have given the extract of belladonna to hundreds of patients, from two months to fifty years of age, and am firmly convinced that it has a greater control over whooping-cough than any other remedy in common use. That, while in a few cases the system did not seem susceptible to its action, in the doses I have prescribed, yet, in nearly all, the disease yielded quickly. It is a safe and efficient remedy for pertussis, in children of any age. It is very remarkable that, after the publications made by Dr. Jackson, in the *Medical Journal*, we should have book after book upon the diseases of children, and the whole host of old and useless remedies for whooping-cough carefully noted, without a word in favor of belladonna.

